# Video-rate all-optical ultrasound imaging

**DOI:** 10.1364/BOE.9.003481

**Published:** 2018-07-02

**Authors:** Erwin J. Alles, Sacha Noimark, Efthymios Maneas, Edward Z. Zhang, Ivan P. Parkin, Paul C. Beard, Adrien E. Desjardins

**Affiliations:** 1 Department of Medical Physics and Biomedical Engineering, University College London, London WC1E 6BT, UK; 2 Wellcome / EPSRC Centre for Surgical and Interventional Sciences, University College London, Charles Bell House, 67-73 Riding House Street, London W1W 7EJ, UK; 3 Materials Chemistry Research Centre, UCL Department of Chemistry, London WC1H 0AJ, UK

**Keywords:** (110.7170) Ultrasound, (170.3880) Medical and biological imaging

## Abstract

All-optical ultrasound imaging, where ultrasound is generated and detected using light, has recently been demonstrated as a viable modality that is inherently insensitive to electromagnetic interference and exhibits wide bandwidths. High-quality 2D and 3D all-optical ultrasound images of tissues have previously been presented; however, to date, long acquisition times (ranging from minutes to hours) have hindered clinical application. Here, we present the first all-optical ultrasound imaging system capable of video-rate, real-time two-dimensional imaging of biological tissue. This was achieved using a spatially extended nano-composite optical ultrasound generator, a highly sensitive fibre-optic acoustic receiver, and eccentric illumination resulting in an acoustic source exhibiting optimal directivity. This source was scanned across a one-dimensional source aperture using a fast galvo mirror, thus enabling the dynamic synthesis of source arrays comprising spatially overlapping sources at non-uniform source separation distances. The resulting system achieved a sustained frame rate of 15 Hz, a dynamic range of 30 dB, a penetration depth of at least 6 mm, a resolution of 75 µm (axial) by 100 µm (lateral), and enabled the dynamics of a pulsating *ex vivo* carotid artery to be captured.

## 1. Introduction

All-optical ultrasound is an emerging imaging modality that shows great promise for biomedical imaging. With this modality, optically absorbing structures convert pulsed or modulated light into ultrasound [1–13] via the photoacoustic effect [14]. Compared to conventional electronic systems comprising piezoelectric or capacitive ultrasound transducer elements, optical transducers have been shown to yield similar or higher pressures and bandwidths [2, 4, 10, 15–18]. When such an optical acoustic source is paired with an optical acoustic receiver, reflected acoustic waves can be captured with an all-optical pulse-echo transducer element that contains no electronics, and hence is MRI compatible and immune to electromagnetic interference. However, despite recent advances, 2D or 3D all-optical ultrasound imaging of biological tissue has been confined to a benchtop imaging modality with image acquisition times ranging from minutes to hours [10, 16, 19–26]. For *in vivo* biomedical imaging, these times need to be decreased by several orders of magnitude to capture dynamic processes in the presence of physiological motion.

To acquire an ultrasonic image, transmission or reception from multiple spatial locations is required, with a 1D array for 2D imaging and a 2D array for 3D imaging. Consequently, conventional electronic imaging probes typically contain arrays of hundreds to thousands of transducers that can be addressed individually in rapid succession. For all-optical ultrasound imaging, previous studies have largely focussed on optimising the performance of individual sources [10, 15, 16, 19–25, 27–30] and receivers [16, 19–21, 31–33], or single-element fibre-optic probes [15, 25, 34, 35]. However, 2D or 3D imaging with a single source and single receiver requires mechanical translation to synthesise an array, which is usually impractical for video-rate speeds required for *in vivo* biomedical imaging. To date, there have been no demonstrations of video-rate 2D all-optical ultrasound imaging of tissue.

In this work, we present a new paradigm for all-optical ultrasound imaging that enables real-time, video-rate 2D ultrasound imaging at a frame rate of 15 Hz. Whereas electronic transducer elements are defined through electrode patterning or physical separation, in this work optical ultrasound sources are shaped and positioned by means of optical confinement achieved using lenses and scanning optics. This allowed for the synthesis of dynamically reconfigurable source arrays of arbitrary geometry [11], thus enabling 1D source arrays exhibiting non-uniform source spacing and reduced image artefacts. In addition, a combination of innovations was used to achieve a sensitivity suitable for imaging weak reflections from deep within biological tissue in real-time. Among these innovations were a centimetre-scale ultrasound generation surface comprising carbon nanotubes and an elastomeric polymer that was suspended in free space, eccentric optical excitation achieved through the use of cylindrical optics, and a high-finesse Fabry-Pérot cavity for ultrasound reception. The sensitivity of the system was demonstrated on different biological tissues, and its real-time dynamic capabilities were demonstrated through the imaging of pulsating blood vessel sections and acoustic contrast agent injections.

## 2. Materials and methods

In all-optical ultrasound imaging, ultrasound is generated *via* the photoacoustic effect within an optical ultrasound generator. Pulsed excitation light is delivered to this generating structure, where it is absorbed and converted into heat. The resulting localised temperature increase results in a pressure increase, which propagates into the surrounding medium as an ultrasonic acoustic wave. As with conventional ultrasound imaging, part of this wave is scattered off acoustic contrasts to an optical ultrasound receiver and recorded as a pulse-echo ultrasound signal (“A-scan”). A-scans detected for source positions spanning the entire source aperture are collected, and finally reconstructed into a 2D pulse-echo (“B-mode”) image. All-optical ultrasound imaging is distinct from photoacoustic imaging: in the former case acoustic signals are generated within a dedicated optical ultrasound generator and reflected off the imaged object; in the latter case the acoustic signals are generated through light absorption *within* the imaged object.

### 2.1. Experimental setup

#### 2.1.1. Excitation light delivery and source array synthesis

**Fig. 1:**
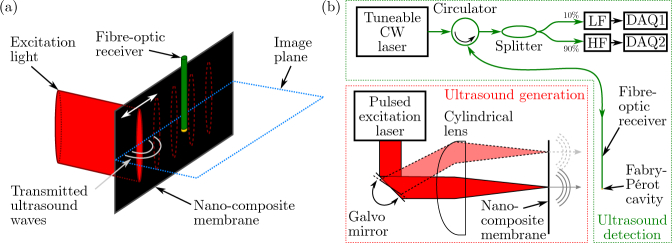
**Optical ultrasound generation and detection. (a)** Using a cylindrical lens, excitation light was delivered to a small, eccentric area of an optically absorbing membrane comprising a nano-composite. Ultrasound was generated photoacoustically in this area, and a linear acoustic aperture was scanned sequentially by translating the focal spot across the membrane using a galvo mirror. **(b)** Schematic of the set-up used to optically generate (red box) and detect (green box) ultrasound. A fibre-optic acoustic receiver, comprising a Fabry-Pérot cavity fabricated at its tip, was interrogated using a tuneable continuous wave laser. Using an optical splitter, 10% of the reflected light was recorded using a low frequency photodiode to record the cavity transfer function in order to identify the resonance wavelength; the remaining 90% was coupled into a high-frequency photodiode to record the acoustic signal. CW: continuous-wave; LF/HF: low-/high-frequency photodiode; DAQ: data acquisition.

Pulsed excitation light (wavelength: 1064 nm, pulse duration: < 5 ns, pulse energy: 76 µJ, pulse repetition rate: 2 kHz, beam diameter: 1.0 mm; FQS-400-1-Y-1064, Elforlight, U.K.) was focussed onto an optically absorbing membrane, where ultrasound was generated photoacoustically within the illuminated region. Using a galvo mirror (GVSM002, Thorlabs, Germany), subsequent light pulses were focussed in different locations along a linear aperture (Fig. 1

), thus creating a sequentially scanned linear acoustic source aperture (width: 15.5 mm). Consecutive excitation light pulses can be delivered to arbitrary positions along the source aperture; hence arbitrary acoustic source array geometries can be synthesised.

Excitation light was focussed in the optical scan direction (lateral direction) to obtain narrow acoustic sources exhibiting low directivity, thus ensuring a high lateral image resolution. To improve the signal-to-noise ratio at depth, a cylindrical lens (focal length: 50 mm; LJ1695RM-C, Thorlabs, Germany) was used to avoid out-of-plane (elevational) optical focussing. This resulted in eccentric acoustic sources that are highly directional in the elevational direction. As a consequence, the transmitted acoustic energy was confined to the image plane, thereby limiting geometrical spreading and the associated signal decay and thus maximising the acoustic signal-to-noise ratio.

#### 2.1.2. Ultrasound generating nano-composite membrane

In this work, a large (approximately 5 cm × 3 cm), optically absorbing membrane was used as optical ultrasound generator. This membrane was suspended backing-free in water to avoid image artefacts due to ringing within the backing. The sound-generating membrane was fabricated by blade coating a 49±6 µm thick layer of multi-walled carbon nanotube-loaded polydimethylsiloxane onto a glass surface covered by a polyimide film. This nano-composite material was previously shown to generate high pressures and bandwidths when applied to the distal ends of optical fibres [10]. The membrane was subsequently peeled off, suspended on an open frame, and mounted a distance of 9 mm away from the wall of the water bath to avoid associated reflection artefacts in the ultrasound images, thus resulting in a maximum image depth of 9 mm.

#### 2.1.3. Ultrasound reception

Image data were acquired by recording the acoustic pulse-echo A-scans for each source location using a single, stationary fibre-optic acoustic receiver comprising a high finesse Fabry-Pérot cavity fabricated on the tip of an optical fibre. This receiver, with a diameter of only 125 µm, achieved a noise equivalent pressure of 40 Pa, exhibited a bandwidth of 80 MHz, and had a nearly omni-directional response for frequencies up to 20 MHz [36]. Combined with the highly efficient acoustic generator and eccentric illumination described above, this receiver achieved a sufficiently high signal-to-noise ratio to detect pulse-echo signals off soft tissue up to a depth of 10 mm without the need for signal averaging.

The fibre-optic receiver was interrogated by measuring the cavity’s reflectivity using a continuous-wave tunable laser (TUNICS T100S-HP, Yenista, France). Using a 90/10% splitter (TW1550R2A2, Thorlabs, Germany), 10% of the reflected light was coupled to and recorded with a low-bandwidth photodiode (PDA20CS-EC, Thorlabs, Germany) and data acquisition board (PCI-6229 + SCB-68A, National Instruments, TX, USA) to determine and tune the interrogation laser to the wavelength corresponding to the peak derivative of the cavity transfer function [37]. The remaining 90% of the light were detected using a broadband photodiode (DET01CFC, Thorlabs, Germany), amplified (+60 dB; DHPVA-200, Femto, Germany), and sampled using a high-speed data acquisition card (125 MSa/s, 14-bit; M4i.4420-x8, Spectrum, Germany) without signal averaging.

The fibre-optic acoustic receiver was placed approximately 1.0 mm in front of the sound-generating membrane, and centered laterally within the image plane. This axial offset of the receiver was set to the minimum distance achievable within the spatial constraints of the setup. Pulse-echo signals acquired across the whole aperture were subsequently reconstructed in real-time to yield a 2D image. A custom LabVIEW script (LabVIEW 2014, National Instruments, TX, USA) was used to control the experimental set-up.

### 2.2. Signal processing and image reconstruction

Recorded A-scans were band-pass filtered (Tukey window with parameter *α* = 8%, pass band: 2 – 15 MHz), and direct cross-talk (ultrasound propagating from each source directly to the receiver) was suppressed through temporal windowing. Prior to reconstruction, power-law time gain compensation was applied to compensate for geometrical and acoustical attenuation. Specifically, a time gain compensated A-scan was computed as *S_i_*(*t*)= *t^α^ Ai(t*), where *A_i_(t*) is the A-scan recorded for source position *i*, *t* is time (s), and the time gain exponent *α* was determined empricially. The A-scans were reconstructed into a 2D image using the delay-and-sum algorithm [38] (which is equivalent to dynamic focussing [39]), assuming omni-directional, point-like sources and receivers and a homogeneous speed of sound. This algorithm yields the image amplitude $I(\vec{r})$ in each image location $\vec{r}$ as

$$I(\vec{r})=\sum_{i=1}^{N}S_{i}\;\left(t=\frac{\left|\vec{r}-{\vec{r}}_{s,i}|+|{\vec{r}}_{d}-\vec{r}\right|}{c}\right),$$ (1)

where ${\vec{r}}_{s,i}$ is the position of source *i*, ${\vec{r}}_{d}$ is the position of the receiver, *c* is the speed of sound, and *N* is the number of acoustic sources. To improve performance, the time delays were computed for the first frame and retained in memory, and the summations were performed using custom pre-compiled software written in C++. Envelope detection along the axial direction was applied upon image display. Image reconstruction was performed using a single core (Core i7-6700 @ 3.4 GHz, Intel, CA, USA) of a PC containing 32 GB of memory, and to enable video-rate display the current frame was reconstructed in parallel with the acquisition of the next frame.

The fibre-optic acoustic receiver was axially offset from the ultrasound-generating membrane, and the arrival times *t_i_* of the direct cross-talk observed for each source position were used to determine both the hydrophone location ${\vec{r}}_{d}=(0,z_{d}=c\cdot \text{min}\;t_{i})$ and source positions ${\vec{r}}_{s,i}=(x_{s,i}=\pm {\left[c^{2}t_{i}^{2}-z_{h}^{2}\right]}^{1/2},0)$, where the sign of *x_s,i_* was given by sgn (*i* − argmin *t_i_*). The speed of sound was computed from the water temperature [40] measured in real-time using a temperature logger (USB-TC01, National Instruments, TX, USA).

### 2.3. Acoustical characterisation

A calibrated needle hydrophone (75 µm, Precision Acoustics, UK) was placed at a distance of 2.7 mm from the ultrasound-generating membrane, and 31 acoustic sources distributed equidistantly along a linear aperture (spacing: 0.5 mm) were sequentially excited. A motorised scan was performed (step size: 25 µm, grid size: 4 mm × 20 mm; MTS50/M-Z8 + TDC001, Thorlabs, Germany) to capture the 31 generated acoustic fields. From each field scan, the A-scan corresponding to the maximum recorded pressure was selected, and the corresponding bandwidth was determined by the −6 dB level of its power spectrum. Each field scan was numerically back-propagated [41] to the membrane to obtain the pressure at the source surface, as well as the spatial extents of the acoustic sources defined by the full-width-at-half-maximum of the surface pressure.

### 2.4. Image artefact reduction through apodisation

Ultrasound source arrays can suffer from both side and grating lobes that deteriorate the image quality. Side lobes are associated with the finite spatial extent of an array, whereas grating lobes occur for equidistantly spaced sources that are separated by more than half the shortest wavelength [42]. For arrays containing conventional piezoelectric or capacitive transducers, where the elements are typically uniformly spaced due to fabrication constraints, amplitude apodisation is commonly applied to suppress side lobes. With amplitude apodisation, weights are applied to the A-scans prior to image reconstruction, thus attenuating sources close to the edge of the array. However, the periodicity of the array remains unaltered, and hence grating lobes are unaffected.

Using a galvo mirror and lens to spatially confine and position excitation light, eccentric acoustic sources can be generated in arbitrary locations. Contrary to arrays of conventional piezoelectric or capacitive transducers, imaging arrays comprising optical ultrasound sources can have an arbitrary geometry, which can be dynamically reconfigured during operation [11]. In addition, as only a single optical acoustic source is excited at a time and each optical ultrasound source is sharply delineated, consecutive sources can exhibit partial spatial overlap without introducing inter-element cross-talk. Consequently, using the free-space all-optical imaging set-up presented here, source arrays can be achieved that exhibit reduced periodicity and an element pitch that is smaller than the optical acoustic source width, resulting in strongly reduced grating lobes. In addition, by decreasing the spatial density of the sources towards the edges of the array, a technique dubbed “source density apodisation” (SDA), side lobes can be reduced in a way similar to conventional amplitude apodisation. Both amplitude and source density apodisation schemes were applied in this work.

#### 2.4.1. Amplitude apodisation

A-scans *S_i_*(*t*) were scaled by weight factor *W* (*i*) which depended on the lateral coordinate of source *i*. Two amplitude apodisation schemes were considered: top-hat apodisation (*W* (*i*) = 1), where effectively no apodisation is applied, and Hamming apodisation $\left(W(i)=0.54-0.46\;\text{cos}\;\left(2\pi \frac{i-1}{N-1}\right)\right)$, which is commonly applied to conventional piezoelectric arrays [43] to suppress artefacts associated with the side and grating lobes typically observed for such arrays. For aperiodic arrays, the Hamming window *W*(*i*) was interpolated to the non-uniform source positions.

#### 2.4.2. Source density apodisation

The flexibility in source positioning achieved with the proposed imaging paradigm allows for arbitrary, aperiodic source array geometries that are difficult to achieve using conventional ultrasound transducers. In addition, the proposed set-up allows for direct comparison of the image quality achieved using different source array geometries implemented on the same hardware. The improvement in image quality obtained using non-uniform array geometries was demonstrated using source density apodisation (SDA), where the spatial density of the sources *D*(*i*) was varied across the linear aperture. Three SDA schemes were considered: top-hat SDA (*D*(*i*) = 1) resulting in uniform source spacing, Hamming SDA [44] $\left(D(i)=0.54-0.46\;\text{cos}\;\left(2\pi \frac{i-1}{N-1}\right)\right)$ resulting in an array where the highest source density is found in the centre of the array, and asin SDA ($x_{s,i}=\frac{2d}{\pi }\;\text{asin}\;\left(2\frac{i-1}{N-1}-1\right)$, where *d* is the full width of the acoustical aperture) resulting in a source density that is approximately halfway in between those obtained using top-hat and Hamming SDA. Source coordinates $x_{s,i}=\int_{1}^{N}D{\left(i\right)}^{-1}di$ were recovered through numerical integration, and scaled to span the full aperture. To improve accuracy, the numerical integration was performed using 1000-fold oversampling. The source separation distances for arrays of 256 sources ranged between 62.8 µm (top-hat SDA), 17.8 − 222 µm (Hamming SDA) and 40.0 – 638 µm (asin SDA); hence significant overlap occurred between subsequent sources (which measured 224 ± 53 µm).

The galvo potentials corresponding to the desired source locations *x_s,i_* were obtained through numerical inversion of the non-linear relationship between galvo potential and source location measured for 256 uniformly sampled galvo potentials spanning the aperture. Images were acquired of a wire phantom comprising two parallel layers of tungsten wires (diameter: 27 µm, spaced 1 mm apart), which exhibited lower echogenicity than typically observed for soft tissue. The first layer was positioned a distance of 3.2 mm away from the sound-generating membrane; the second layer at a distance of 6.2 mm. Reconstructed images measured 16 mm × 8 mm with a pixel size of 10 µm × 10 µm, and a time gain exponent of *α* = 0.5 was used. Pulse-echo data were acquired for 32, 64, 128, 256, 512, and 1024 source locations, using all three SDA schemes. Each of these eighteen data sets was reconstructed using both top-hat and Hamming amplitude apodisation. The spatial resolution was determined by the full-width-at-half-maximum spatial extent of the image of the nearest on-axis wire.

### 2.5. Tissue imaging

#### 2.5.1. Static imaging

An adult zebrafish [line Tg(fli1a-nEGFP)] obtained post-mortem and fixed in 2% paraformaldehyde was bonded (using cyanoacrylate) to a thin film (150 µm thick polyvinyl chloride (PVC); “cling film”) stretched around an empty frame. The zebrafish was submerged in water and positioned at a distance of approximately 2.4 mm from the ultrasound-generating membrane. Using two orthogonal motorised stages (MTS50/M-Z8 + TDC001, Thorlabs, Germany), the head of the zebrafish was imaged *ex vivo* in either a single coronal plane through the eye, or in a stack of 26 transverse planes spaced 0.5 mm apart. Each all-optical ultrasound image (size: 16 mm × 8 mm; pixel size: 50 µm × 25 µm) was obtained using 1024 source locations, asin source density apodisation, Hamming amplitude apodisation, and a time gain exponent of *α* = 1.5. To compare the achieved image quality, a conventional clinical ultrasound scanner (SonixMDP, Analogic Ultrasound, MA, USA) and high-frequency probe (L40-8/12, Vermon, France) were used in free-hand operation to acquire images in approximately the same image planes.

#### 2.5.2. Dynamic imaging

A section of healthy, *ex vivo* swine carotid artery was bonded (using cyanoacrylate) to a rigid acrylic backing. The proximal end of the vessel was connected to a 30 ml syringe through a section of syringe tubing; the distal end was either clamped shut or left open. The vessel was placed approximately 2.5 mm away from the sound-generating membrane, and centered in the lateral direction. The vessel phantom was imaged at fifteen frames per second using 128 source locations, asin source density apodisation, top-hat amplitude apodisation, and a time gain exponent of *α* = 0 (equivalent to not applying time gain compensation). Each frame measured 16 mm × 9 mm at a pixel size of 50 µm × 25 µm.

Two experiments were performed. In the first experiment, the distal end of the vessel was clamped shut, and the vessel was pressurised and released thrice by manual compression of the syringe. In the second experiment, the distal end of the vessel was left open and the syringe was filled with a solution of glass bubbles (concentration: 10 g/l, diameter: 20 – 80 µm; Glass Bubbles type S32, 3M, MN, USA) in water. For both experiments, an M-mode image was generated by concatenating the central image line extracted from each frame.

## 3. Results

### 3.1. Ultrasound generation performance

**Fig. 2:**
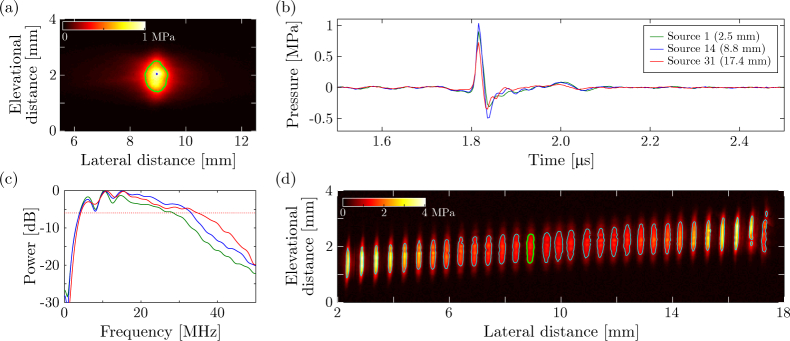
**Characterisation of the acoustic sources. (a)** Peak acoustic pressure generated by a single source measured across a plane placed 2.7 mm away from the ultrasound-generating membrane. The green contour indicates the full-width-at-half-maximum (FWHM) of the acoustic pressure; the blue dot indicates the location where the corresponding pressure data (shown in blue) of panels (b-c) were recorded. **(b)** Temporal pressure profiles emitted by three optical ultrasound sources positioned at lateral co-ordinates of 2.5 mm (green), 8.8 mm (blue; corresponding to the pressure field displayed in panel (a)) and 17.4 mm (red). These profiles were recorded at a distance of 2.7 mm and measured directly in front of each source. **(c)** Power spectra (normalised to 0 dB) of the three temporal pressure profiles displayed in panel (b). The dotted red line corresponds to the −6 dB level used to determine the acoustic bandwidth. **(d)** Compound image of the peak pressure at the surface of the ultrasound-generating membrane. The contours correspond to the FWHM obtained for each of the 31 sequentially addressed sources, and are representative of the size of the acoustical sources. The green contour corresponds to the source measured in panel (a).

The ultrasound generation performance was characterised in terms of pressure amplitude, bandwidth and source dimensions for 31 distinct and equidistantly spaced optical acoustic sources. For all sources spread across the aperture, uniform pressures and temporal pressure profiles (Fig. 2(a–b)

; 0.977 ± 0.061 MPa) and bandwidths (Fig. 2(c); 27.1 ± 2.17 MHz) were recorded at a distance of 2.7 mm from the sound-generating membrane, and their small standard deviations confirmed the spatial uniformity of the performance of the sound-generating membrane.

Upon numerical back-propagation, narrow source widths (Fig. 2(d); 224 ± 53 µm) were obtained in the lateral (in-plane) direction. This confirmed the efficacy of the cylindrical lens in laterally confining the optical excitation light, and demonstrated mechanical cross-talk within the nano-composite membrane was negligible. As a trend, the optical acoustic sources were widest in the centre of the aperture. A slight misalignment in optics resulted in an elevational offset of the acoustic sources that increased linearly with their lateral co-ordinates. In the elevational (out-of-plane) direction, no optical focussing occurred, as confirmed by the elevational source extent (1.10 mm ± 82 µm) that is similar to the excitation laser beam diameter of 1.0 mm.

The elevational extent of the acoustic field observed at a distance of 2.7 mm (Fig. 2(a)) was identical to the elevational extent at the ultrasound generating membrane surface (Fig. 2(d)). This observation, together with simulations performed for circular and eccentric ultrasound sources (data not shown), confirms that the acoustic sources emit pressure fields that are elevationally confined to the image plane, thereby obviating the need for an additional focussing lens.

### 3.2. Uniform versus non-uniform source arrays

**Fig. 3:**
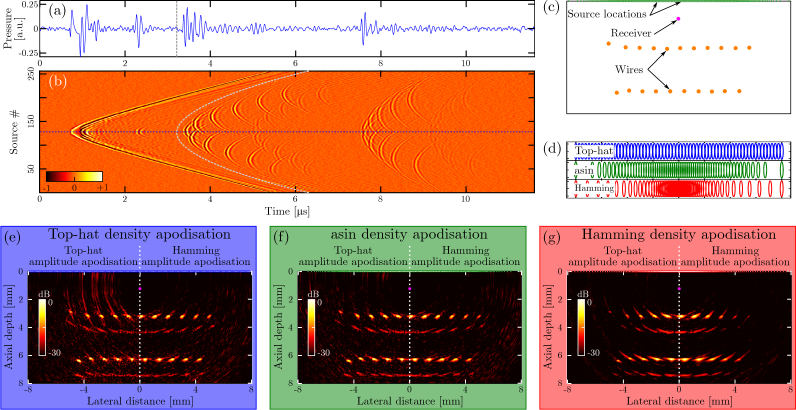
**Artefact reduction through source density apodisation. (a)** A-scan for an optical ultrasound source positioned in the centre of the aperture. Pulse-echo data were acquired of a phantom comprising two layers of tungsten wires (diameter: 27 µm). No signal averaging was performed to acquire these data. **(b)** Pulse-echo B-scan across the entire source aperture. Pulse-echo events of the shallow and deep layers occur at times 3.5 < *t* < 6.5 µs and 7.5 < *t* < 9.5 µs, respectively. The events prior to the cut-off times indicated by the gray dashed curve correspond to sound waves that propagated from the optical acoustic sources directly to the receiver (“direct cross-talk”); this cross-talk was removed by setting all samples prior to the cut-off indicated in grey to zero. **(c)** Schematic of the experimental geometry, where 256 sources were positioned along a linear aperture, and the fibre-optic receiver was centered laterally and offset axially. Two layers of wires were placed perpendicular to the image plane. **(d)** Schematic of the source locations corresponding to top-hat, asin and Hamming source density apodisation. To improve visibility, the locations of only 64 sources are shown. Horizontal and vertical ticks correspond to 2 mm and 0.5 mm, respectively. **(e–g)** All-optical ultrasound images of the wire phantom obtained using top-hat, asin and Hamming source density apodisation, respectively. Images were reconstructed using both top-hat (left) and Hamming (right) amplitude apodisation.

To demonstrate the efficacy of SDA, images of a wire phantom were obtained using multiple SDA schemes (Fig. 3

). Three schemes were considered: top-hat SDA, where the source density was constant; Hamming SDA, where the source density was given by the Hamming function; and “asin” SDA, where the source locations were given by the trigonometric asin function. Images of an acoustically weakly reflective wire phantom confirmed that applying amplitude apodisation to a uniformly spaced array (Fig. 3(e)) does indeed suppress many image artefacts, and that applying only SDA achieves a similar improvement in image quality (Fig. 3(f–g)). The highest image contrast was achieved when both Hamming amplitude apodisation and Hamming SDA were applied; however, for this combination an inhomogeneous image intensity and lower spatial resolution were observed (lateral: 100 µm for Hamming SDA versus 90 µm for top-hat SDA; axial: 75 µm for Hamming SDA versus 70 µm for top-hat SDA).

When these experiments were repeated using different numbers of sources, the same observations could be made (data not shown). Notably, compared to a uniform array combined with Hamming amplitude apodisation, an array obtained with Hamming SDA combined with Hamming amplitude apodisation yielded a similar image contrast using only approximately half the number of sources. Thus, through the application of SDA the image frame rate can almost be doubled, while retaining the image contrast.

### 3.3. Tissue imaging

**Fig. 4:**
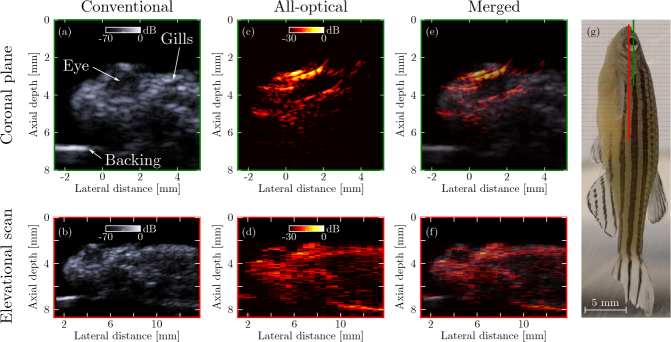
**Ultrasound images of an *ex vivo* zebrafish**. Images were obtained with both a conventional high-frequency piezoelectric array probe **(a–b)** and the presented all-optical set-up **(c–d)**. Conventional and all-optical images were merged **(e–f)** to facilitate comparison. Panels (a) and (c) were obtained from single images along the coronal plane through the eye; panel (d) was extracted from a stack of 26 images acquired in transverse planes spaced 0.5 mm apart. Panel **(g)** shows the location of the single coronal plane (green line), as well as the individual transverse planes (red dotted lines) and the extracted image plane (solid red line).

Post-mortem all-optical ultrasound imaging of a zebrafish (Fig. 4

) yielded images at a dynamic range of 30 dB, an artefact level of −25 dB, and a penetration depth of at least 6 mm (limited here by the size of the zebrafish). In these images, anatomical features (skin, eye, gill) were visualised at a contrast level and resolution similar to those obtained using a conventional high-frequency probe, albeit at a reduced dynamic range. The overly bright area above the eye (Fig. 4(c)) is caused by the specular reflection off the skin being recorded only in that area. In addition, “wing-shaped” artefacts are visible in the same image. The prominence of both the specular reflection and the artefacts stem from the use of only a single point of ultrasound reception. All-optical ultrasound images were recorded along the coronal plane through the eye, and were acquired either directly or extracted from a stack of transverse images (Fig. 4(d)). In the latter case, the achieved elevational resolution of approximately 0.5 mm confirmed the acoustic elevational focussing achieved through the use of eccentric illumination.

**Fig. 5:**
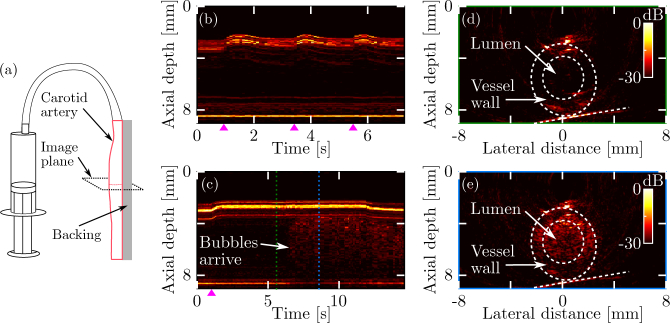
**Dynamic 2D all-optical ultrasound imaging of an *ex vivo* swine carotid artery. (a)** Schematic of the phantom geometry. A section of carotid artery was fixed to a rigid backing and connected to a syringe. Both syringe and artery were filled with water. **(b)** M-mode image of the line through the centre of the artery. With the distal end of the artery clamped shut, images were continuously recorded (frame rate: 15 Hz) while the syringe was manually compressed and released thrice. **(c)** M-mode image obtained during flushing of the artery with water loaded with glass bubbles. **(d–e)** B-mode images obtained before and after the arrival of the bubble bolus, respectively. These images were acquired at the time points indicated by the green and blue dashed lines in panel (c). The real-time, video-rate reconstructed B-mode images corresponding to the compression and flushing experiments can be viewed in Visualization 1 and Visualization 2, respectively. Time points corresponding to pressure onset are indicated ( 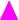).

Video-rate all-optical ultrasound images of a dynamic phantom comprising an *ex vivo* swine carotid artery were acquired and displayed at a frame rate of 15 Hz. Manual pressurisation of a vessel segment fixed to a rigid backing was performed to mimic pulsatile blood flow, and resulted in deformation of the vessel that was clearly visible in both M-mode and B-mode all-optical ultrasound images (Fig. 5(b)

). In a separate experiment, microbubbles were used as acoustic contrast agents and flushed through the vessel. In this experiment, both the vessel deformation and the arrival of these microbubbles were readily visualised (Fig. 5(c–e)). In both experiments, a dynamic range of 30 dB was achieved, at an artefact level below −25 dB. In these images, the sides of the vessel are only partially visualised due to limited-view artefacts caused by the finite dimensions of the source array. In addition, image artefacts similar to those observed in Figs. 3 and 4 were present. Videos of the images acquired during these two experiments can be viewed in Visualization 1 and Visualization 2.

## 4. Discussion and conclusion

In this work, an all-optical ultrasound imaging paradigm is presented that for the first time allows for real-time, video-rate 2D imaging of soft tissue. This was achieved through the use of a highly efficient optical ultrasound generator, a highly sensitive fibre-optic receiver, eccentric illumination resulting in acoustic sources exhibiting elevationally constrained acoustic radiation patterns, a modest excitation pulse repetition rate and pulse energy, fast sequential scanning of a source aperture using a galvo mirror exhibiting low inertia, and source arrays exhibiting non-uniform source spacing and partially overlapping sources. A wire phantom was used to determine the resolution of the system (axial: 75 µm, lateral: 100 µm), and the penetration depth (up to at least 6 mm) and dynamic range (up to 30 dB) were determined using tissue samples. In addition, the high frame rate (15 Hz) was used to capture the dynamics of a pulsating vessel phantom and the injection of an acoustic contrast agent.

All-optical ultrasound has the potential to overcome several fundamental limitations of its electronic counterparts. Electronic ultrasound transducers typically derive their sensitivity from mechanical resonance at a fixed frequency and bandwidth, resulting in a fixed spatial resolution and penetration depth. In addition, electronic ultrasound imaging probes typically comprise transducer elements that are spatially fixed and arranged in a periodic array, which can result in image artefacts [42]. As a result, electronic imaging probes are optimised for specific applications, and typically multiple probes are required for versatility. In contrast, optical ultrasound sources do not rely on resonance, and hence can be tuned to either high resolution imaging or large imaging depth through temporal modulation of the excitation light [45]. In addition, the position and spatial confinement of optically generated ultrasound sources can be varied dynamically using optical methods [11] to avoid imaging artefacts and tailor the source aperture to a wider range of applications.

Compared to conventional ultrasound imaging arrays comprising piezoelectric or capacitive transducers, the all-optical ultrasound set-up achieved a lower dynamic range of 30 dB that was limited by comparatively high image artefact levels (the “wing-shaped” artefacts observed in Fig. 3 and 4) and relatively low signal-to-noise ratios. The image artefact levels were due to the use of a low number of acoustic sources and a single receiver, combined with the delay-and-sum image reconstruction algorithm, and could be reduced in various ways. First, multiple fibre-optic ultrasound receivers can be distributed across the aperture, similar to conventional electronic linear arrays comprising a multitude of electronic transducers that can each both transmit and receive acoustic signals. This way, the back-scattered acoustic field can be recorded in multiple locations in parallel, resulting in the detection of a larger fraction of the back-scattered energy at the same frame rate. This will both decrease the artefact levels and alleviate the observed limited-view artefacts, at the expense of a significant increase in experimental complexity and cost. Second, subsequent ultrasound sources can exhibit spatial overlap without exhibiting cross-talk, and hence an arbitrary number of sources can be positioned within the aperture. Consequently, the image quality could be improved by using more source locations. However, currently the image acquisition and reconstruction rate is limited by the computational cost of the reconstruction and the inertia of the galvo mirror, and hence faster mirrors (for instance resonant galvo mirrors) and parallelised reconstruction algorithms would be required to accommodate the higher pulse repetition rate required to maintain high frame rates. Third, the artefact levels could be further reduced by implementing different source density apodisation (SDA) schemes. While the schemes studied in this work achieved a significant improvement in image quality, alternative SDA schemes might yield even further improvements. Further research is required to determine the most effective SDA schemes. Finally, different image reconstruction algorithms that better exploit the spatial coherence of consecutive A-scans, such as Delay, Multiply and Sum (DMAS) [46] or Short-Lag Spatial Coherence (SLSC) [47], might result in reduced artefact levels.

The dynamic range of the images could be improved further by increasing the signal-to-noise ratio of the acquired data. It has previously been shown [9] that the presence of a rigid backing can improve the efficiency of optical ultrasound sources, thus improving the A-scan signal-to-noise ratio. However, the presence of a rigid backing introduced spurious ringing artefacts (data not shown) that decreased the acoustic bandwidth and the image quality; hence in this work an ultrasound generating membrane was suspended in free-space. Alternatively, increasing the optical fluence would result in larger pressures to be generated in the ultrasound-generating membrane. However, as the fluence used in this work (38 mJ/cm^2^) approached the damage threshold of the membrane (approximately 175 mJ/cm^2^; data not shown), the pressure increase achievable using a pulsed light source is limited. However, alternative optical excitation schemes, such as coded excitations or temporally modulated pulses [45], could be employed to deliver more optical energy over an extended period of time without exceeding the optical damage threshold.

The optical ultrasound sources distributed across the source aperture were found to consistently generate a wide bandwidth ranging between 4 – 31 MHz. However, the lateral extent of the sources (224 ± 53 µm) was too large to fully exploit this bandwidth: the sources were highly directional for higher frequencies, and thus only the lower frequencies contributed to the images. Hence, in this work, signals were hence band-pass filtered between 2 – 15 MHz, which limited the achieved resolution. To utilise the full generated bandwidth, acoustic sources of reduced lateral extent should be used. These can be achieved through a change in optics, for instance using a lens with a shorter focal length. However, to avoid damaging the ultrasound-generating membrane, the pulse energy should be decreased when decreasing the optical spot size, thus reducing the generated pressure amplitude and signal-to-noise ratio. In addition, in the setup presented in this work, the lens focusses excitation light onto the ultrasound-generating membrane through the transparent wall of a water bath and a layer of water, resulting in a wall-reflection artefact in the images at a depth of approximately 3.5 cm. Decreasing the focal length reduces the maximum distance between the water bath wall and the sound-generating membrane, and thus will limit the maximum imaging depth. Ultimately, optical and acoustical scattering within the nano-composite membrane will limit the minimum lateral extent of the acoustic sources.

In the 2D all-optical ultrasound imaging paradigm presented here, the lateral field of view was limited by the lateral extent of the acoustical aperture (15.5 mm), which in turn was limited by the exit pupil of the optics. Thus, through a change in optics a wider source aperture could be achieved. However, to fully exploit a wider aperture, the optical alignment needs to be improved to avoid the elevational offset of the sources observed in Fig. 2. In addition, the fibre-optic acoustic receiver was positioned within the image plane and placed in front of the sound-generating membrane. As a result, ultrasound reflected off the generator membrane resulted in the additional ghost images observed in Fig. 3. These spurious reflections could be limited by positioning the receiver in the plane of the sound-generating membrane, at the expense of a reduced acoustical aperture due to optical shadowing. Alternatively, the ringing could be removed through source deconvolution [48].

The galvo mirror used in this work actually comprised two orthogonal angle-adjustable mirrors, of which one was kept fixed. However, in principle this galvo mirror allows for arbitrary scanning of excitation light in 2D. Using the presented centimetre-scale nano-composite membrane, a 2D acoustic source aperture is hence readily generated that could be used for 3D imaging. If the cylindrical lens were replaced with a spherical lens, an isotropic resolution of approximately 100 µm could be achieved using the same imaging paradigm, at a frame rate of up to 1 Hz. However, as the resulting optical focus is much smaller, the pulse energy of the excitation light would need to be significantly reduced to avoid optical damage to the ultrasound-generating membrane.

To the authors’ knowledge, the study presented here is the first demonstration of video-rate, real-time all-optical ultrasound imaging of clinically relevant tissue. The imaging target was fully submerged in water, which was done to simplify the experimental setup; in principle, acoustic coupling is only required between the imaging target and the optical acoustic sources and receiver. In future configurations, water surrounding the probe could therefore be replaced with acoustic coupling gel applied to only the surface of the imaging target, as is customary in routine clinical practice. Alternatively, a bundle of optical waveguides could be used to obtain a long, flexible imaging probe allowing for free-hand operation similar to current clinical ultrasound probes, or miniaturised for endoscopic applications [11]. As the electrical and metal components can be spatially separated from the acoustic sources and receiver, an all-optical ultrasound probe can be made insensitive to electromagnetic interference. This will enable the application of 2D or 3D ultrasound imaging in electromagnetically harsh or sensitive contexts, such as real-time monitoring of radiofrequency ablation, electrophysiology and neuromodulation. In addition, the application of all-optical ultrasound imaging within an MRI scanner will provide clinicians with concurrent, multi-scale imaging yielding supplementary information during MRI-guided interventional procedures, such as high-precision instrument localisation, video-rate real-time monitoring of MRI-guided high-intensity focussed ultrasound (HIFU) treatment, and visualising microbubble mediated drug delivery. Finally, the adaptability of the frequency, bandwidth and geometry of optical ultrasound sources will allow for seamless, dynamic tailoring of an imaging system to the imaging target to optimise the image quality, frame rate and resolution.
